# Strabismic imaging for correcting in-plane motion distortion in scanning imaging instruments and rolling shutter cameras

**DOI:** 10.1117/1.ap.8.2.026016

**Published:** 2026-04-08

**Authors:** Gastón A. Ayubi, Karteek Kunala, Bartlomiej Kowalski, Aubrey Hargrave, Alfredo Dubra

**Affiliations:** Byers Eye Institute, Stanford University, Palo Alto, California, United States

**Keywords:** rolling shutter effect, motion distortion, scanning microscopy

## Abstract

Images acquired with point-scanning instruments, line-scanning instruments, and rolling shutter cameras are distorted by motion of the instrument or the scene. This distortion arises from the sequential capture of pixels or rows of pixels and is known as the rolling shutter effect. Here, we demonstrate the correction of rolling shutter distortion caused by in-plane motion with a dual-imaging technique inspired by strabismus, an eye misalignment condition. When the velocity observed in pairs of synchronously captured and misaligned images can be considered constant within each image row or column, the distortion estimation problem can be formulated as a system of linear equations describing the displacement of image strips along the slow scanning direction or as an optimization problem that maximizes the similarity between the two distortion-corrected images. Both methods can be modified to correct motion distortion caused by in-plane rotation and scaling changes. Strabismic image pairs, acquired with a point-scanning instrument and a dual-rolling shutter camera setup, are used to demonstrate both approaches.

## 1 Introduction

**Fig. 1:**
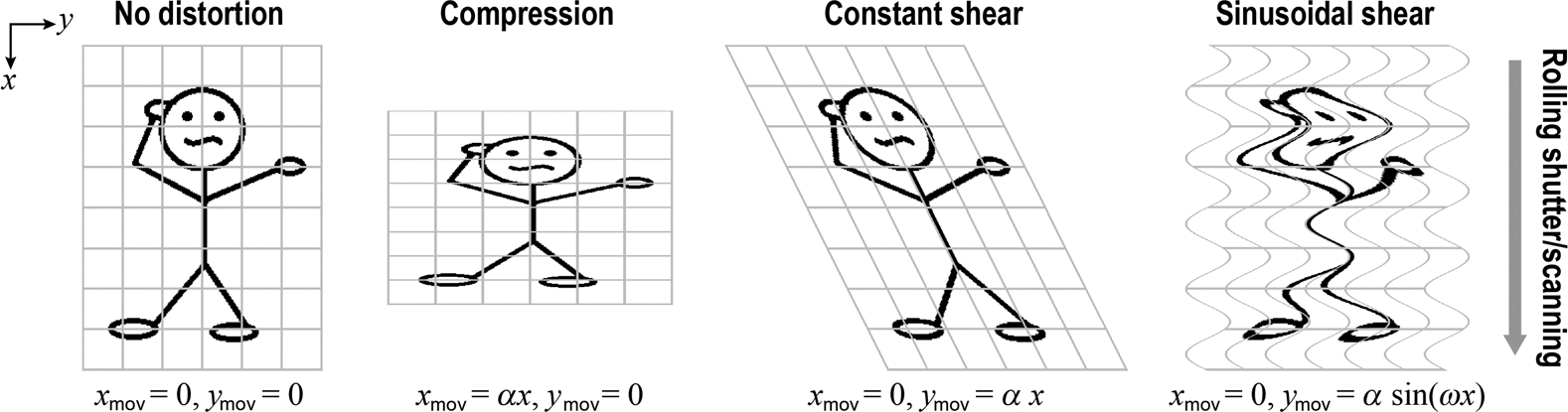
Depiction of image distortion due to $x_{\text{mov}}$ and $y_{\text{mov}}$ motion during the capture of images with scanning instruments or rolling shutter cameras moving from top to bottom (α and ω constants).

Scanning imaging instruments and rolling shutter cameras are ubiquitous in scientific, medical, and industrial applications as well as in end-user products. Point-scanning confocal and multiphoton microscopes have transformed biological and biomedical research by providing optical sectioning of intact live tissue and organisms.^1,2^ Light sheet microscopes, which also provide optical sectioning, deliver high imaging rates by employing rolling shutter cameras.^3,4^ Point- and line-scanning medical imaging instruments are widely used in diagnostic and treatment-monitoring applications, such as endoscopy^5–11^ and ophthalmoscopy.^12–18^ Rolling shutter cameras can be found among many other applications in smartphones, webcams, computer vision systems, robotics, unmanned aerial vehicles, and autonomous vehicles. Images captured with all these devices can exhibit motion distortion, a phenomenon commonly known as the rolling shutter effect (see Fig. 1).

Strategies for estimating motion in rolling shutter instruments can be broadly classified into four categories: motion model or prior knowledge-based approaches, multi-image capture, employing neural networks, and hybrid methods. Examples of these approaches include making assumptions about constant velocity or smooth velocity variations across sequences of consecutive frames.^19–21^ Other methods use information from orientation and position sensors synchronized with the rolling shutter to correct motion distortion^22^ or infer motion from assumptions about the geometry of image features, such as straight lines and vanishing directions.^23^ Among the most recent neural-network-based approaches, Cao et al.^24^ estimated distortion across image sequences through spatiotemporal modeling derived from the sequences themselves, whereas more traditional approaches rely on training sets of images—a strategy with limited generalizability, particularly in research settings where new sample types, imaging modalities, and pathologies are encountered regularly.^25–27^ Also recently, combinations of assumptions about velocity, learning, and the creation of undistorted images by combining two or more sequentially captured images are emerging, seeking to improve robustness, accuracy, and/or broadening applicability.^28–31^ Image distortion has also been estimated using two or more images captured with an additional synchronized device operating with opposite rolling shutter directions and registrations using landmarks^32^ or neural networks. A motion-correction approach now used in commercial point-scanning ophthalmic optical coherence tomography systems involves co-registering two or more sequential images acquired with orthogonal fast-scanning directions to mitigate motion distortion.^33,34^

Here, we demonstrate the measurement and correction of in-plane relative motion between imaging devices and the object or scene within the field of view, using a dual-imaging approach inspired by strabismus (eye misalignment). Distortion is estimated from two synchronous imaging channels that share the same scanning or rolling shutter direction but have offset fields of view. Strabismic imaging, as demonstrated below, represents an evolution of Luo et al.’s^35^ dual-wavelength imaging, eliminating longitudinal and transverse chromatic aberration, removing the need for an additional light source, increasing motion sampling, and reducing distortion errors at the image ends. The proposed algorithms for estimating distortion also improve upon those by Lee et al.^9,11^ and Harlow et al.,^10^ both of which rely on identifying image landmarks for registration. The proposed strabismic imaging addresses major limitations of most current rolling shutter correction methods, most notably the rapid variation of the motion velocity (amplitude and direction) during the acquisition of a single image and the need for training datasets. The former is simply due to the fact that, without assumptions of constant or smooth and monotonic variation of velocity across frames or specific assumptions about the presence of geometrical features within the field of view, the distortion estimation is an ill-posed mathematical problem.

## 2 Theory

### 2.1 Strabismic Imaging Principle

**Fig. 2:**
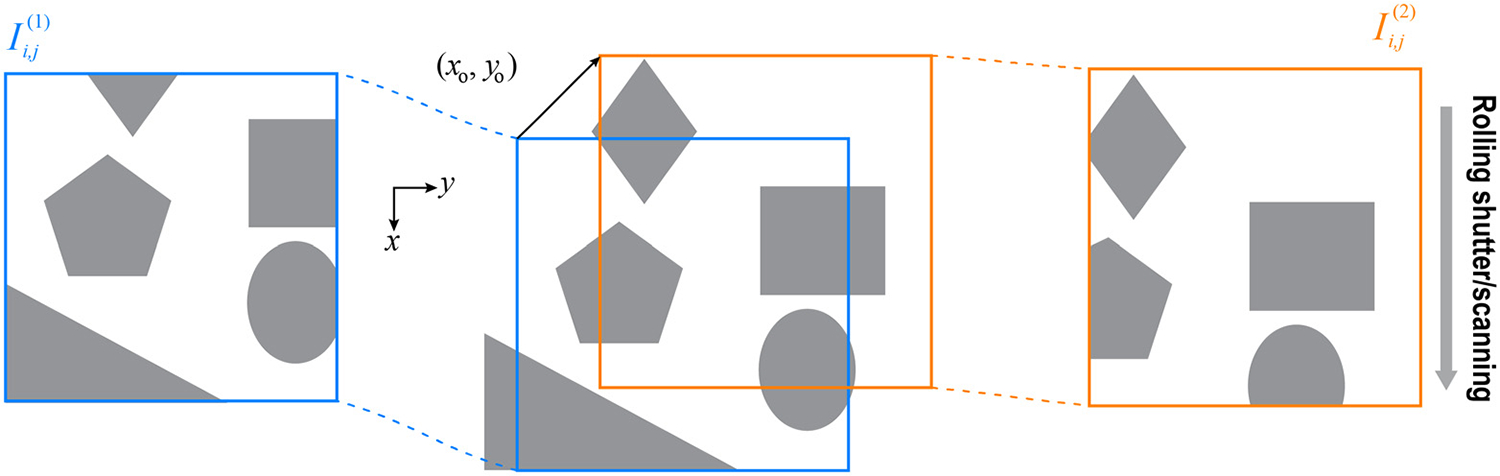
Strabismic imaging for estimating motion distortion in scanning instruments and rolling shutter cameras, in which two simultaneously captured images from the same scene have offset fields of view.

Let us consider a single- or multi-channel point-scanning instrument, line-scanning instrument, or a dual-rolling shutter camera system that captures two synchronized images whose fields of view are offset along the (slow) scanning direction, which we assume here runs from the top to the bottom of the images. We also assume that these images have been corrected for scanning velocity variation and/or optical distortion,^36–38^ such that the image pixels lie on a regular rectangular grid parallel to the axes of a Cartesian coordinate system. The pixel values of the primary strabismic image $I_{i,j}^{\left(1\right)}$ and secondary image $I_{i,j}^{\left(2\right)}$ of scene intensity S(x,y) can then be described as

(1) $$I_{i,j}^{\left(1\right)}=S\left(i\delta x-x_{\text{mov}}(i,j),j\delta y-y_{\text{mov}}(i,j)\right)$$

and

(2) $$I_{i,j}^{\left(2\right)}=S\left(i\delta x-x_{\text{mov}}(i,j)-x_{\text{o}},j\delta y-y_{\text{mov}}(i,j)-y_{\text{o}}\right),$$

where δx and δy are the spacing between pixel rows and columns, respectively; $x_{\text{mov}}$ and $y_{\text{mov}}$ are functions that describe the scene movement in relation to the instrument; and $\left(x_{\text{o}},y_{\text{o}}\right)$ is the spatial offset among fields of view (see Fig. 2). In line-scanning instruments and rolling shutter cameras, all the pixels in each row are captured simultaneously, and thus, the motion is only a function of the row index i. In point-scanning instruments, however, pixels along each row are captured sequentially, but often sufficiently fast to consider the motion negligible within each row. When this is the case, strabismic images for all three types of instruments can be described as

(3) $$I_{i,j}^{\left(1\right)}=S\left(i-x_{\text{mov}}(i),j-y_{\text{mov}}(i)\right)$$

and

(4) $$I_{i,j}^{\left(2\right)}=S\left(i-x_{\text{mov}}(i)-x_{\text{o}},j-y_{\text{mov}}(i)-y_{\text{o}}\right),$$

where for convenience and without loss of generality, we express all distances in units of pixel separation (i.e., δx=δy=1).

As will be shown later, estimating motion functions from a single pair of strabismic images can be formulated as either two independent sets of coupled finite difference equations derived from coregistering image strip pairs or as an optimization problem in which the images are iteratively distorted to either minimize a cost function or to maximize a similarity metric. In both approaches, a horizontal offset is acceptable provided it remains substantially below 50% of the image width, whereas vertical overlap is required, and must be substantially less than 50% of the image height. These offsets must be experimentally measured and incorporated in the motion estimation to avoid misinterpreting them as artifacts caused by a constant velocity assumption. The offsets can be determined by co-registering a pair of strabismic images from a stationary object, for example, by maximizing their normalized weighted cross-correlation (NWCC) or their zero-mean normalized weighted cross-correlation (ZNWCC). The weighting is an option that enables the use of images with non-rectangular boundaries and the prioritization of image regions based on desired characteristics, such as signal-to-noise ratio.^39^ The registration accuracy can be improved by upsampling the strabismic images, upsampling the ZNWCC function, or applying iterative optimization methods.^40^

We now introduce two formulations for describing and estimating motion functions that describe in-plane translations. Both formulations can be extended to account for rotation and scaling changes during the image capture, as we discuss later.

### 2.2 Linear Algebra Solutions

Each image from the scanning instruments considered here can be viewed as consisting of strips formed by contiguous rows. By coregistering strip pairs, one from each image, we derive a set of finite-difference equations for the motion functions, which can then be solved by computing a generalized inverse of the system matrix.^41–43^ In this way, a strip centered on row $i_{1}$ with radius M can be written as

(5) $$I_{i_{1}+i^{\prime },j}^{\left(1\right)}=S\left(i_{1}+i^{\prime }-x_{\text{mov}}\left(i_{1}+i^{\prime }\right),j-y_{\text{mov}}\left(i_{1}+i^{\prime }\right)\right),$$

with $i^{\prime }=-M,\ldots ,M$, whereas a strip in the second image centered on $i_{2}$ would be

(6) $$I_{i_{2}+i^{\prime },j}^{\left(2\right)}=S\left(i_{2}+i^{\prime }-x_{\text{mov}}\left(i_{2}+i^{\prime }\right)-x_{\text{o}},j-y_{\text{mov}}\left(i_{2}+i^{\prime }\right)-y_{\text{o}}\right).$$

The relative strip shifts $\Delta x_{\text{strip}}\left(i_{1},i_{2}\right)$ and $\Delta y_{\text{strip}}\left(i_{1},i_{2}\right)$ can be estimated using registration methods, such as the maximization of the strips NWCC or ZNWCC functions,^39^ both of which are feature-agnostic. Here, we use the latter because it is immune to gain and offset differences between the image pair. The accuracy of the strip coregistration using the ZNWCC is limited by distortion within the strips, which we address through multiple cycles of motion estimation and correction. After each iteration, the image strips become less and less distorted to the point that Eqs. (5) and (6) no longer depend on $i^{\prime }$, allowing us to define strip displacement as

(7) $$\Delta x_{\text{strip}}\left(i_{1},i_{2}\right)=i_{1}-x_{\text{mov}}\left(i_{1}\right)-i_{2}+x_{\text{mov}}\left(i_{2}\right)+x_{\text{o}}$$

and

(8) $$\Delta y_{\text{strip}}\left(i_{1},i_{2}\right)=-y_{\text{mov}}\left(i_{1}\right)+y_{\text{mov}}\left(i_{2}\right)+y_{\text{o}},$$

which can be rearranged as

(9) $$x_{\text{mov}}\left(i_{2}\right)-x_{\text{mov}}\left(i_{1}\right)=\Delta x_{\text{strip}}\left(i_{1},i_{2}\right)+i_{2}-i_{1}-x_{\text{o}}$$

and

(10) $$y_{\text{mov}}\left(i_{2}\right)-y_{\text{mov}}\left(i_{1}\right)=\Delta y_{\text{strip}}\left(i_{1},i_{2}\right)-y_{\text{o}}.$$

**Fig. 3:**
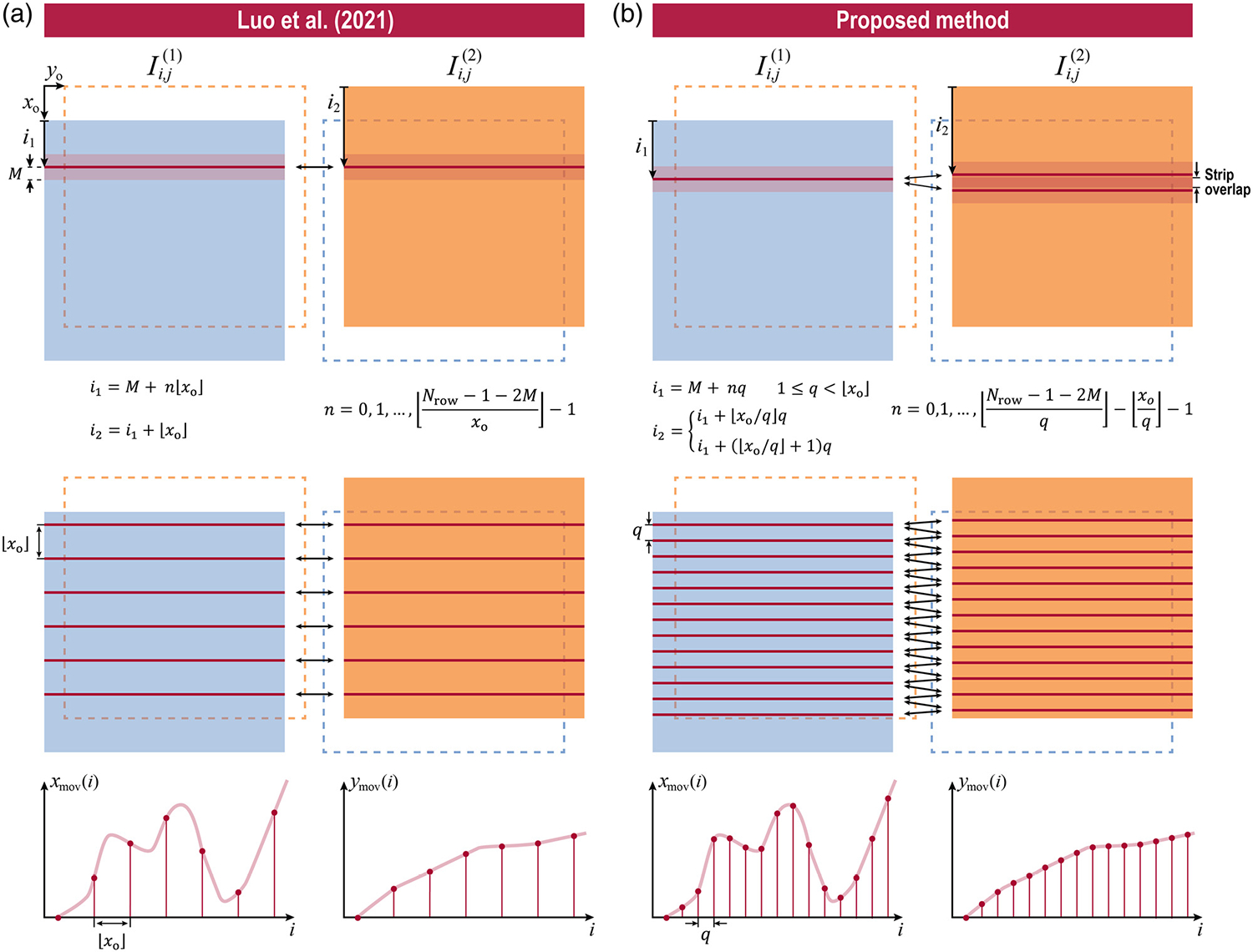
Luo et al.^35^ (a) and proposed (b) motion estimation from simultaneously captured strabismic image pairs (blue and orange rectangles) by co-registering image strip pairs (transparent red rectangles and dark red lines indicating their centers) with row indices $i_{1}$ and $i_{2}$ along the (slow) scanning or rolling shutter direction. In Luo et al.’s method, the strip pairs are naturally coupled through spacing that matches the vertical image offset (rounded off by the floor function). In the proposed method, higher motion sampling is achieved through an additional set of strips to create a coupled set of equations (black arrows between strip centers), with the plots at the bottom depicting motion functions and their sampling.

In their original work, Luo et al.^35^ used a set of strips defined by $i_{1}=M+n\left\lfloor x_{\text{o}}\right\rfloor$, with $n=0,1,\ldots ,\left(N_{\text{row}}-1-2M\right)/\left\lfloor x_{\text{o}}\right\rfloor -1,\lfloor \rfloor$ denotes the floor function, and $i_{2}=i_{1}+\left\lfloor x_{\text{o}}\right\rfloor$. In this way, the vertical separation between strip pairs is the vertical offset among the strabismic imaging channels, which could be too coarse, as depicted on the left side of Fig. 3. This choice of strip centers leads to two undetermined sets of coupled equations, one for $x_{\text{mov}}$ and one for $y_{\text{mov}}$, with one free variable each that shifts the motion function in relation to the origin of the system of coordinates. Therefore, in what follows, we set the first value of both motion functions to zero, effectively making the image’s top-left pixel the origin of coordinates.

To improve Luo et al.’s method, we propose sets of strips with uniform but arbitrary vertical separation q such that $1\leq q<\left\lfloor x_{\text{o}}\right\rfloor$ to increase the motion function sampling as desired, also generating two sets of coupled equations as before. We achieve this by selecting strips in the first strabismic image with central row $i_{1}=M+nq$ with $n=0,1,\ldots ,\left\lfloor \left(N_{\text{row}}-1-2M\right)/\right.q\rfloor -\left\lfloor x_{\text{o}}/q\right\rfloor -1$, each of which is coregistered with two strips in the second strabismic image with central rows $i_{2}=i_{1}+\left\lfloor x_{\text{o}}/q\right\rfloor q$ and $i_{2}=i_{1}+\left(\left\lfloor x_{\text{o}}/q\right\rfloor +1\right)q$, as shown on the right side of Fig. 3. It is critical that the height and vertical separation of each pair of image strips used to create an equation have adequate overlap for coregistration.

The resulting set of motion function finite differences derived from Eq. (9) can be expressed as the matrix equations

(11) $$\left[\begin{array}{c} \Delta x_{\text{strip}}\left(M,M+\left\lfloor \frac{x_{0}}{q}\right\rfloor q\right)+\left\lfloor \frac{x_{0}}{q}\right\rfloor q-x_{0}\\ \vdots \\ \Delta x_{\text{strip}}\left(M+nq,M+\left(\left\lfloor \frac{x_{0}}{q}q\right\rfloor +n\right)q\right)+\left\lfloor \frac{x_{0}}{q}\right\rfloor q-x_{0}\\ \vdots \\ \Delta x_{\text{strip}}\left(M,M+\left(\left\lfloor \frac{x_{0}}{q}\right\rfloor +1\right)q\right)+\left(\left\lfloor \frac{x_{0}}{q}\right\rfloor +1\right)q-x_{0}\\ \vdots \\ \Delta x_{\text{strip}}\left(M+nq,M+\left(\left\lfloor \frac{x_{0}}{q}\right\rfloor +1+n\right)q\right)+\left(\left\lfloor \frac{x_{0}}{q}\right\rfloor +1\right)q-x_{0}\\ \vdots \end{array}\right]=\left[\begin{array}{ccccc} -1 & \leftarrow \left\lfloor \frac{x_{0}}{q}\right\rfloor \to & 1 & & \\ & \ddots & & \ddots & \\ & & -1 & \leftarrow \left\lfloor \frac{x_{0}}{q}\right\rfloor \to & 1\\ -1 & \leftarrow \left\lfloor \frac{x_{0}}{q}\right\rfloor +1\to & 1 & & \\ & \ddots & & \ddots & \\ & & -1 & \leftarrow \left\lfloor \frac{x_{0}}{q}\right\rfloor +1\to & 1 \end{array}\right]\times \left[\begin{array}{c} x_{\text{mov}}(M)\\ \vdots \\ x_{\text{mov}}\left(M+\left\lfloor \frac{x_{0}}{q}\right\rfloor q\right)\\ x_{\text{mov}}\left(M+\left(\left\lfloor \frac{x_{0}}{q}\right\rfloor +1\right)q\right)\\ \vdots \\ x_{\text{mov}}(M+nq)\\ \vdots \\ x_{\text{mov}}\left(M+\left(\left\lfloor \frac{x_{0}}{q}\right\rfloor +n\right)q\right)\\ x_{\text{mov}}\left(M+\left(\left\lfloor \frac{x_{0}}{q}\right\rfloor +1+n\right)q\right)\\ \vdots \end{array}\right]$$

and

(12) $$\left[\begin{array}{c} \Delta y_{\text{strip}}\left(M,M+\left\lfloor \frac{x_{0}}{q}\right\rfloor q\right)-y_{0}\\ \vdots \\ \Delta y_{\text{strip}}\left(M+nq,M+\left(\left\lfloor \frac{x_{0}}{q}\right\rfloor +n\right)q\right)-y_{0}\\ \vdots \\ \Delta y_{\text{strip}}\left(M,M+\left(\left\lfloor \frac{x_{0}}{q}\right\rfloor +1\right)q\right)-y_{0}\\ \vdots \\ \Delta y_{\text{strip}}\left(M+nq,M+\left(\left\lfloor \frac{x_{0}}{q}\right\rfloor +1+n\right)q\right)-y_{0}\\ \vdots \end{array}\right]\;=\left[\begin{array}{ccccc} -1 & \leftarrow \left\lfloor \frac{x_{0}}{q}\right\rfloor \to & 1 & & \\ & \ddots & & \ddots & \\ & & -1 & \leftarrow \left\lfloor \frac{x_{0}}{q}\right\rfloor \to & 1\\ -1 & \leftarrow \left\lfloor \frac{x_{0}}{q}\right\rfloor +1\to & 1 & & \\ & \ddots & & \ddots & \\ & & -1 & \leftarrow \left\lfloor \frac{x_{0}}{q}\right\rfloor +1\to & 1 \end{array}\right]\times \;\left[\begin{array}{c} y_{\text{mov}}(M)\\ \vdots \\ y_{\text{mov}}\left(M+\left\lfloor \frac{x_{0}}{q}\right\rfloor q\right)\\ y_{\text{mov}}\left(M+\left(\left\lfloor \frac{x_{0}}{q}\right\rfloor +1\right)q\right)\\ \vdots \\ y_{\text{mov}}(M+nq)\\ \vdots \\ y_{\text{mov}}\left(M+\left(\left\lfloor \frac{x_{0}}{q}\right\rfloor +n\right)q\right)\\ y_{\text{mov}}\left(M+\left(\left\lfloor \frac{x_{0}}{q}\right\rfloor +1+n\right)q\right)\\ \vdots \end{array}\right],$$

in which the dark bold font indicates the separation among matrix elements, with all matrix elements not associated with the differences being zero. Solutions to this system of equations can be found by calculating generalized inverse matrices, such as the Moore–Penrose pseudoinverse, which we use here both because it provides least-squares solutions and its availability in widely used programming languages.^44–46^ If the strip spacing q is larger than one pixel, then the resulting sequence of motion function values can be interpolated as needed. Irrespective of the value of q, the estimated $x_{\text{mov}}$ and $y_{\text{mov}}$ values should be low pass filtered to mitigate coregistration errors due to discretization and image distortion within each strip. As mentioned earlier, errors in the motion functions caused by distortion within each strip can be mitigated by iterative measurement and correction of image distortion by composing the estimated motion function values as described next.

Let us index consecutive motion-function estimates by Roman numerals: I denotes the initial images, II denotes the first distortion-corrected images, III denotes the second distortion-corrected images, and so on. For a pixel with initial indices $\left(i_{\text{I}},j_{\text{I}}\right)$, we have

(13) $$i_{\text{II}}=x_{{\text{mov}}_{\text{I}\to \text{II}}}\left(i_{\text{I}}\right)+i_{\text{I}}$$

and

(14) $$i_{\text{III}}=x_{{\text{mov}}_{\text{II→II}}}\left(i_{\text{II}}\right)+i_{\text{II}}.$$

When combined, these equations give

(15) $$i_{\text{III}}=x_{{\text{mov}}_{\text{II}\to \text{III}}}\left(x_{{\text{mov}}_{\text{I}\to \text{II}}}\left(i_{\text{I}}\right)+i_{\text{I}}\right)+x_{{\text{mov}}_{\text{I}\to \text{II}}}\left(i_{\text{I}}\right)+i_{\text{I}},$$

which allows writing the composed motion function as

(16) $$x_{{\text{mov}}_{\text{I}\to \text{III}}}\left(i_{\text{I}}\right)=x_{{\text{mov}}_{\text{II}\to \text{III}}}\left(x_{{\text{mov}}_{\text{I}\to \text{II}}}\left(i_{\text{I}}\right)+i_{\text{I}}\right)+x_{{\text{mov}}_{\text{I}\to \text{II}}}\left(i_{\text{I}}\right),$$

and, similarly

(17) $$y_{{\text{mov}}_{\text{I}\to \text{III}}}\left(i_{\text{I}}\right)=y_{{\text{mov}}_{\text{II}\to \text{III}}}\left(x_{{\text{mov}}_{\text{I}\to \text{II}}}\left(i_{\text{I}}\right)+i_{\text{I}}\right)+y_{{\text{mov}}_{\text{I}\to \text{II}}}\left(i_{\text{I}}\right).$$

This compounding can be repeated to calculate the distortion of corrected images from the original images (i.e., with full distortion) through a single pixel interpolation, rather than multiple interpolations that would degrade image fidelity.

### 2.3 Optimization Solutions

Motion estimation in strabismic imaging can also be formulated as an optimization problem, in which the opposite motion functions (i.e., $x_{\text{mov}}^{\prime }$ and $y_{\text{mov}}^{\prime }$) are sought to minimize the difference among the overlapping image regions,

(18) $$x_{\text{mov}}\left(i\right),y_{\text{mov}}\left(i\right)=\underset{x_{\text{mov}}^{\prime }\left(i\right),y_{\text{mov}}^{\prime }\left(i\right)}{\text{arg}\;\text{min}}\left\{{\sum_{i^{\prime },j^{\prime }\epsilon I_{i^{\prime },j^{\prime }}^{\left(1\right)}\cap I_{i^{\prime },j^{\prime }}^{\left(2\right)}}\left(I_{i+x_{\text{mov}\left(i\right),j+y_{\text{mov}}^{\prime }\left(i\right)}^{\prime }}^{\left(1\right)}-I_{i+x_{\text{mov}}^{\prime }\left(i\right)+x_{\text{o}},j+y_{\text{mov}}^{\prime }\left(i\right)+y_{\text{o}}}^{\left(2\right)}\right)}^{2}\right\},$$

or to maximize an image similarity metric, such as the ZNWCC, over the overlapping image regions,

(19) $$x_{\text{mov}}\left(i\right),y_{\text{mov}}\left(i\right)=\underset{x_{\text{mov}}^{\prime }\left(i\right),y_{\text{mov}}^{\prime }\left(i\right)}{\text{arg}\;\text{max}}\left\{{\text{ZNWCC}}_{I_{i^{\prime },j^{\prime }}^{\left(1\right)}\cap I_{i^{\prime },j^{\prime }}^{\left(2\right)}}\left(I_{i+x_{\text{mov}}^{\prime }\left(i\right),j+y_{\text{mov}}^{\prime }\left(i\right)}^{\left(1\right)},I_{i+x_{\text{mov}}^{\prime }\left(i\right)+x_{\text{o}},j+y_{\text{mov}}^{\prime }\left(i\right)+y_{\text{o}}}^{\left(2\right)}\right)\right\}.$$

Either of these undetermined equations can be solved by imposing smoothness constraints and/or regularization terms, as is commonly done in optical flow problems.^47^

**Fig. 4:**
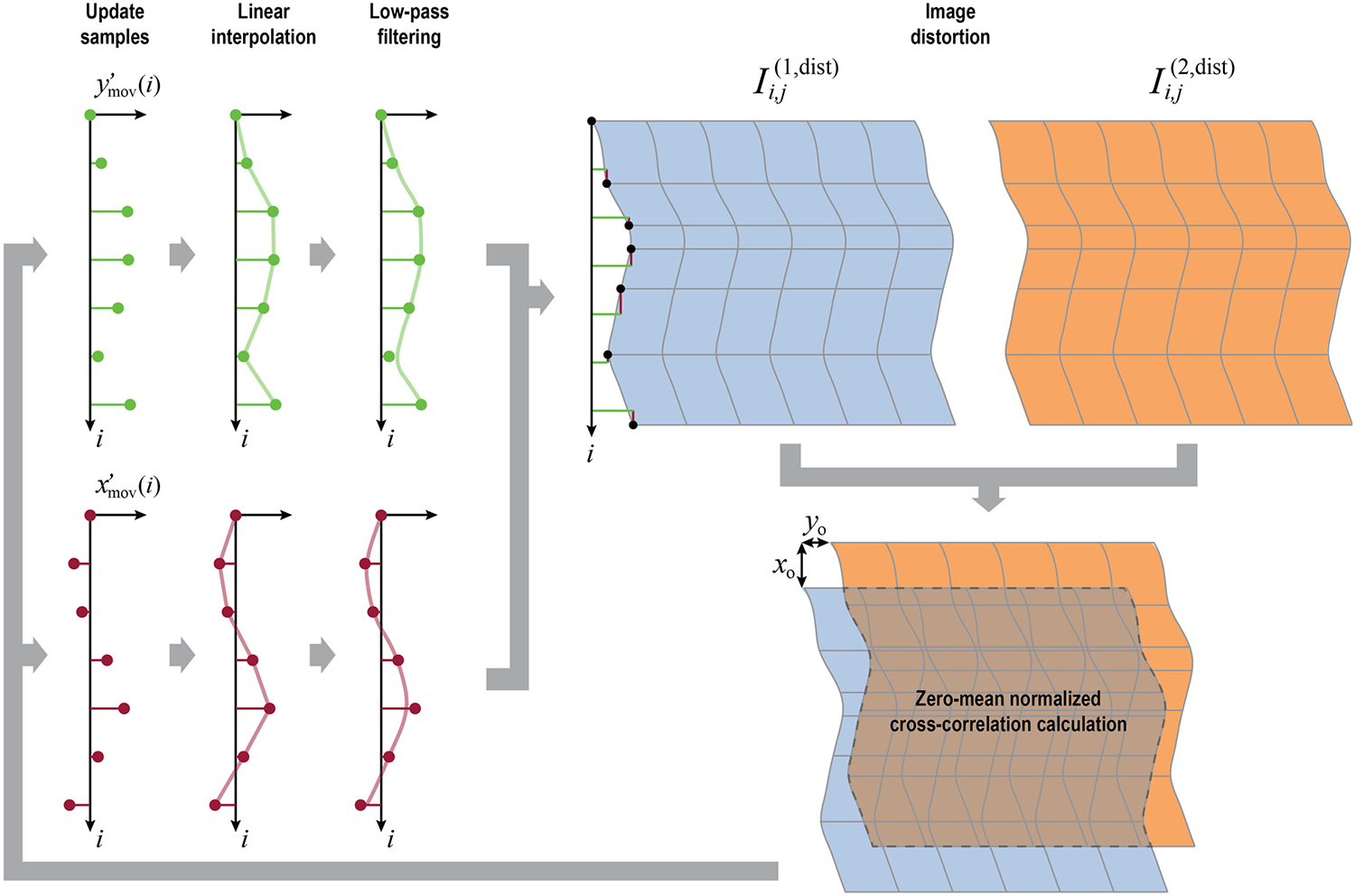
Proposed optimization algorithm for estimating motion distortion in strabismic image pairs (blue and orange shapes), by applying iterative horizontal and vertical deformations along the image rows to maximize the ZNWCC between the image pair.

Here, we implemented the ZNWCC maximization as depicted in Fig. 4, by varying a number of uniformly spaced $x_{\text{mov}}^{\prime }$ and $y_{\text{mov}}^{\prime }$ samples, with their first and last samples assigned to the first and last image rows, respectively. As our smoothness/regularization constraint, these values were linearly interpolated across all image rows and then smoothed with a Gaussian filter whose standard deviation equals the initial sample spacing. To mitigate filtering artifacts near the top and bottom of the image, the $x_{\text{mov}}^{\prime }$ and $y_{\text{mov}}^{\prime }$ domains were extended by mirror reflection, inversion, and offsetting to ensure continuity of the motion functions and their first derivatives at the image top and bottom. During the iterative optimization process, the $x_{\text{mov}}^{\prime }$ and $y_{\text{mov}}^{\prime }$ samples were varied one at a time through the modified version of Powell’s minimization algorithm^48^ in Python SciPy module, version 1.15.2. The optimization convergence was facilitated by providing the motion functions estimates from the linear algebra approach as the initial sample values, reducing calculation time and ensuring that the resulting solutions are equal to or better than those from the linear algebra method. As with the linear algebra approach described earlier, the initial values of both motion functions are set to zero, without loss of generality.

## 3 Instruments

Two custom instruments were used to demonstrate motion estimation from strabismic image pairs: a point-scanning instrument and a dual-rolling shutter camera setup, with the latter being equivalent to a line-scanning instrument.

### 3.1 Point-Scanning Ophthalmoscope

Pairs of strabismic images were captured using a custom adaptive optics scanning light ophthalmoscope (AOSLO), which can be thought of as a reflectance confocal microscope with a horizontal optical scanner resonating at 13.8 KHz and a non-resonant vertical scanner operating at 14.9 Hz.^49^ This instrument was modified by replacing the beam splitter that couples the source and detector optics in the reflection and transmission paths with an uncoated glass substrate 2.8 mm thick with a 5-arcmin angle among its surfaces. This glass wedge reflects two laterally shifted beams with comparable intensity and a small tilt among them, chosen so that the beams overlap at pupil conjugate planes and are separated in image space by 0.3 deg. The offset between the primary and secondary fields of view was adjusted by rotating the glass wedge. An additional light detector was used to capture the secondary strabismic image synchronously with the primary detector at a rate of 40 MHz.

### 3.2 Dual-Rolling Shutter Camera Setup

**Fig. 5:**
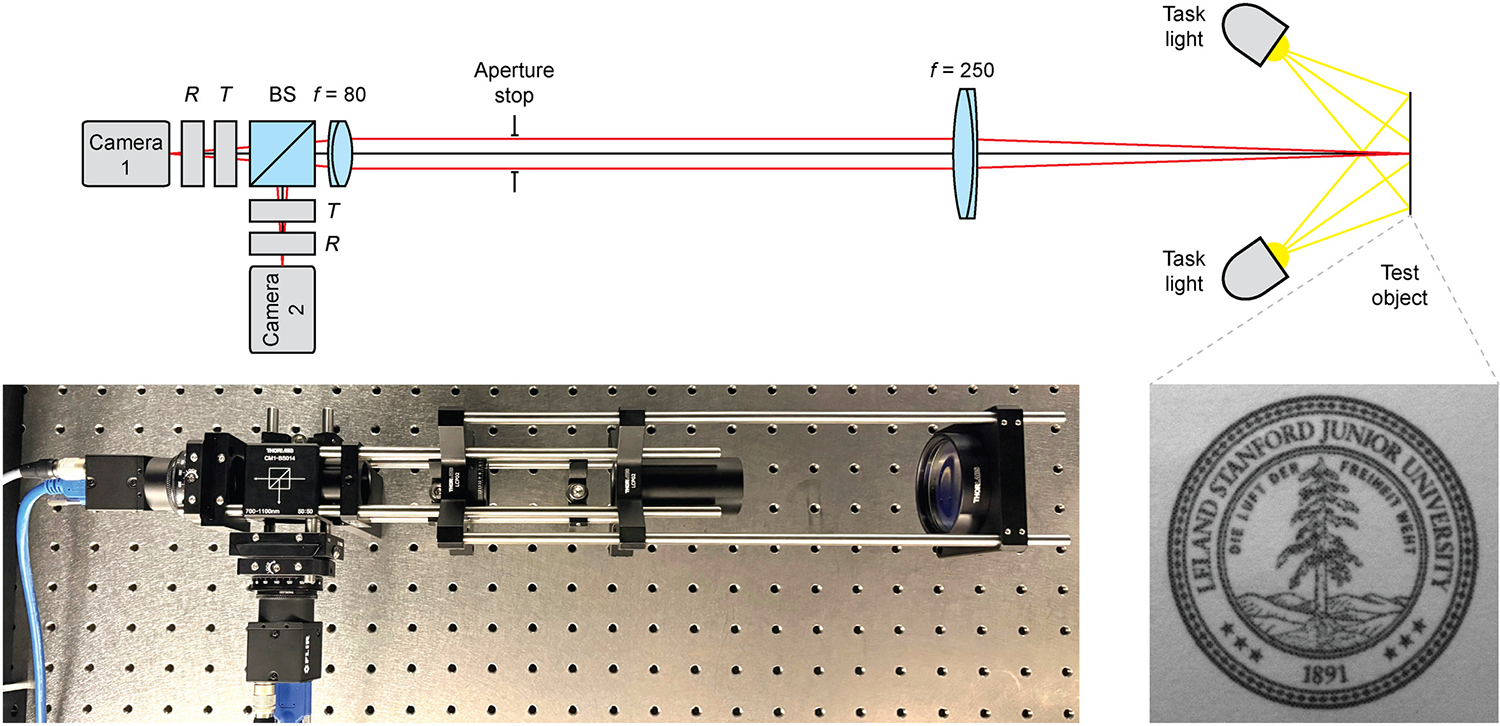
Diagram and picture of strabismic imaging setup using two synchronized rolling shutter cameras (R and T are rotation and vertical translation stages, respectively; BS is a beam splitting cube; and f denotes the focal length in units of mm).

Pairs of strabismic images were captured using a custom setup with two synchronous monochromatic rolling shutter cameras (BFS-U3-63S4M-C by Teledyne FLIR, Wilsonville, Oregon, United States), under temporally and spatially incoherent illumination from two task lights, as depicted in Fig. 5. The optical paths of the two cameras were combined using a beam splitting cube, such that they shared the same lenses. Images from the camera in the beam splitter reflection path were flipped left–right prior to strabismic image processing. The cameras were operated with 2 × 2 pixel binning and 0.1-ms exposure.

## 4 Human Subject

One subject with no known ocular pathology was recruited for retinal imaging with the custom adaptive optics ophthalmoscope. Research procedures and informed consent followed the tenets of the Declaration of Helsinki and were approved by the institutional review board of Stanford University. Prior to imaging, pupil dilation and cycloplegia were achieved with one drop of 2.5% phenylephrine and one drop of 1% tropicamide. A bite-bar with a soft dental impression was attached to a manually operated three-axis translation stage to align and stabilize the subject during imaging.

## 5 Experiments

After capturing strabismic image pairs with the AOSLO and dual-rolling shutter camera setup, we estimated initial motion functions for each pair using the linear algebra approach and subsequently refined them via the optimization method. The quality of the motion distortion correction was quantified as the maximum of the ZNWCC between the distortion corrected image pair, a normalized similarity metric that attains a value of one only when the two images are identical over the overlapping regions. Departures from unity should not always be interpreted as poor distortion correction; they can arise from differences among imaging channels, such as vignetting and blur variation across the field of view.

**Fig. 6:**
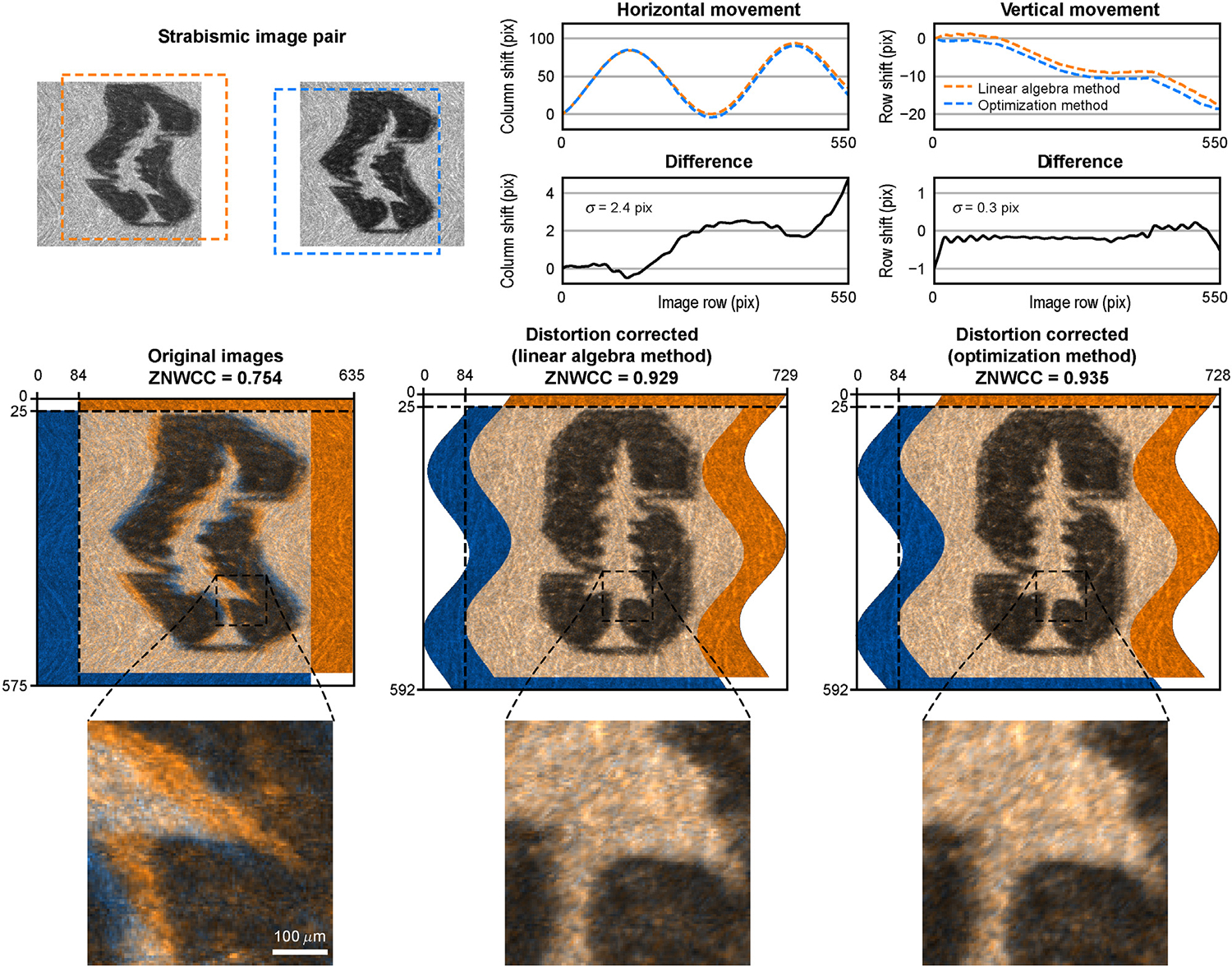
Strabismic image pairs of a Stanford logo acquired with a point-scanning instrument (slow scanning from top to bottom) distorted by left-to-right sinusoidal motion and their respective outlines showing their relative positions (top left) and estimated motion (top right). Overlapped scanning and optical distortion-corrected images (bottom left) and corresponding motion corrected images. For this image pair, the linear algebra approach was implemented as a single iteration with one 9-pixel high strip per line (i.e., q=1) and refined by three optimization iterations with 8.6-pixel sample spacing.

To facilitate the visualization of differences among overlapping images, we display them in blue and orange, complementary colors to accommodate common color vision deficiencies. When overlapping pixels have similar values, they appear monochromatic (black-grey-white), and when they differ, they appear colored. An overall orange or blue tint, as in Fig. 6, indicates differences in detector gain and/or offset.

### 5.1 Point-Scanning Ophthalmoscope

A printed Stanford University logo was imaged with the AOSLO while inducing sinusoidal motion mostly along the horizontal direction with ~25 and ~50 pixels in amplitude. The results from the strabismic image processing for the strongest motion are shown in Fig. 6, demonstrating the correction of the sinusoidally varying horizontal shear and vertical compression/expansion. The small differences among the motion functions indicate a small but non-negligible (>1 pixel) improvement of the optimization over the linear algebra solution.

**Fig. 7:**
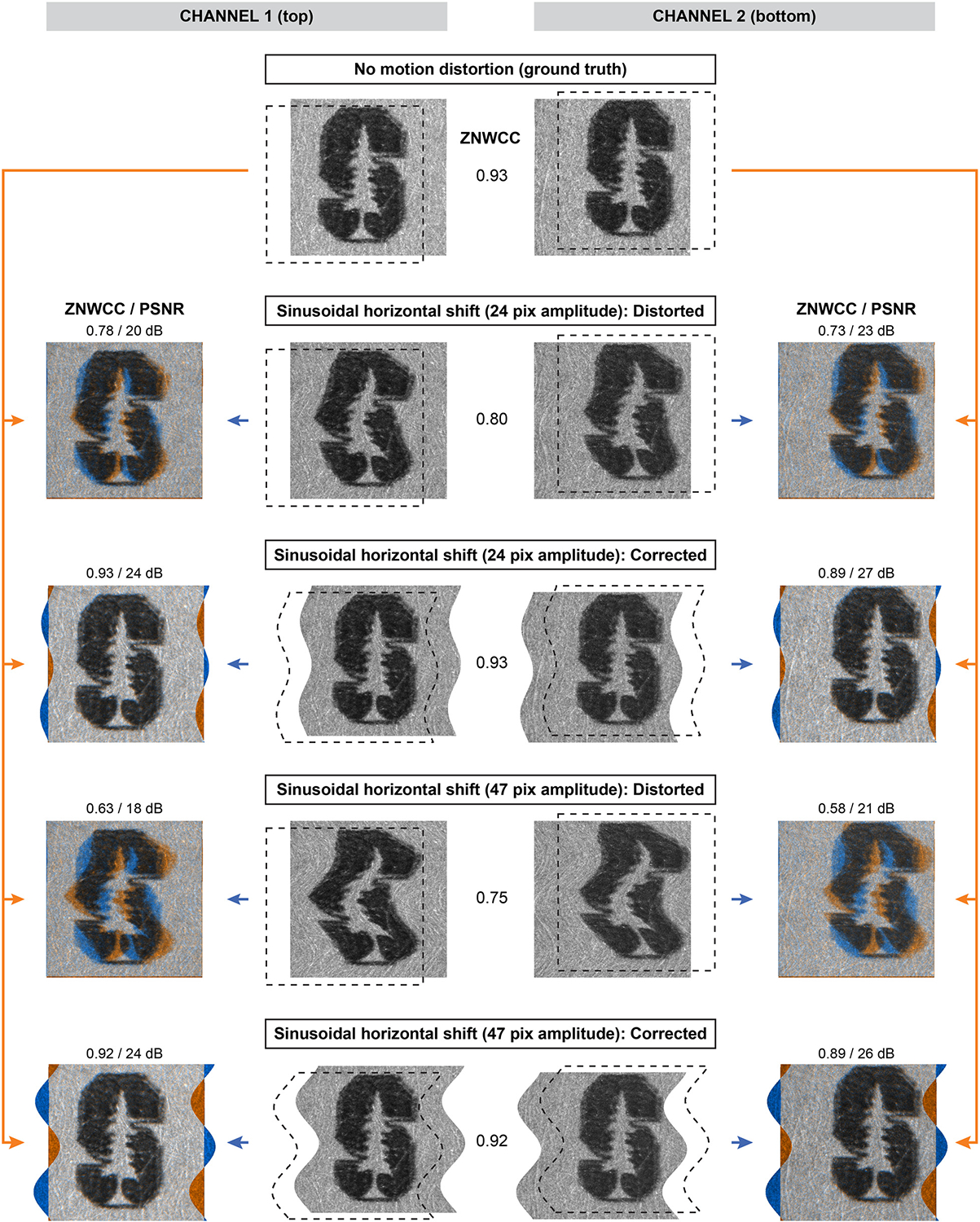
Strabismic image pairs of a Stanford logo acquired with a point-scanning instrument (slow scanning from top to bottom) and their respective outlines showing their relative positions. The top row shows the ground truth (no motion), the second and fourth rows show left-to-right sinusoidal motion distortion, and the third and fifth rows show the corresponding distortion-corrected images (linear algebra first, one iteration with 9-pixel high strips, q=1, and optimization approach later, three iterations with 8.6-pixel sample spacing). The numerical values along the image central column are the ZNWCC values for the respective strabismic image pairs, whereas the values on top of the overlapping images are the ZNWCC and the PSNR.

The images in Fig. 7 compare distortion-corrected images with ground-truth images collected with no motion for both imaging channels. The ZNWCC between corresponding strabismic image pairs is 0.93 in the no-motion condition but remains below unity due to the differences among imaging channels. Image pairs with motion distortion and initial ZNWCC values of 0.80 and 0.75 improve after distortion correction to 0.93 and 0.92, respectively, approaching the ground-truth level and illustrating the effectiveness of the proposed approach. This improvement is consistent with per-channel comparisons of distortion-corrected images against their corresponding ground-truth images, whether quantified by ZNWCC or by peak signal-to-noise ratio (PSNR).^50^

**Fig. 8:**
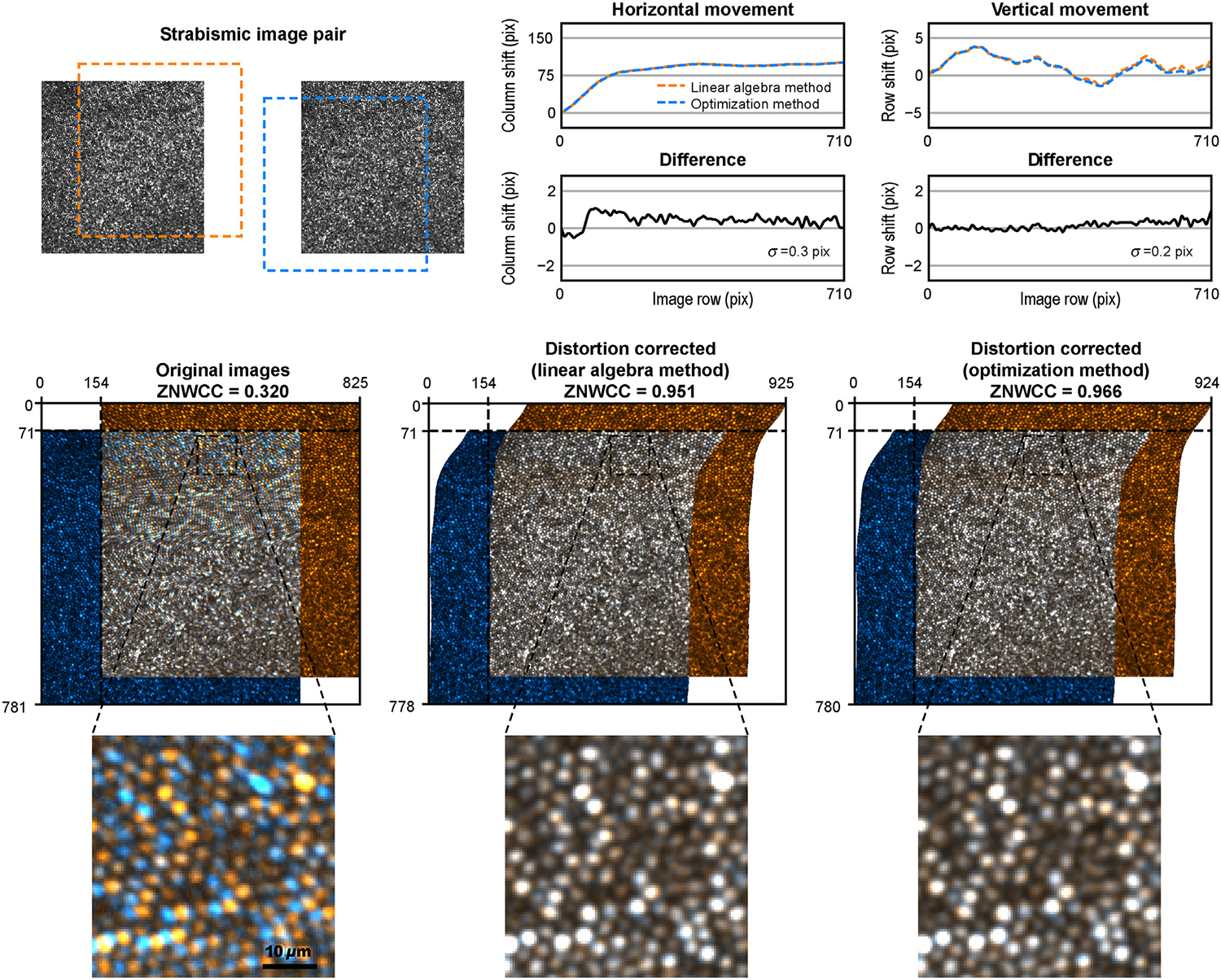
Strabismic reflectance image pair of a human photoreceptor mosaic acquired with a point-scanning confocal ophthalmoscope (slow scanning from top to bottom), their respective outlines showing their relative positions (top left) and the estimated motion (top right). The images are shown before and after correction of the distortion caused by involuntary fixational eye movement, with the linear algebra approach first (one iteration with 5-pixel high strip, q=1), and then refined by three optimization iterations with 8.6-pixel sample spacing.

**Fig. 9:**
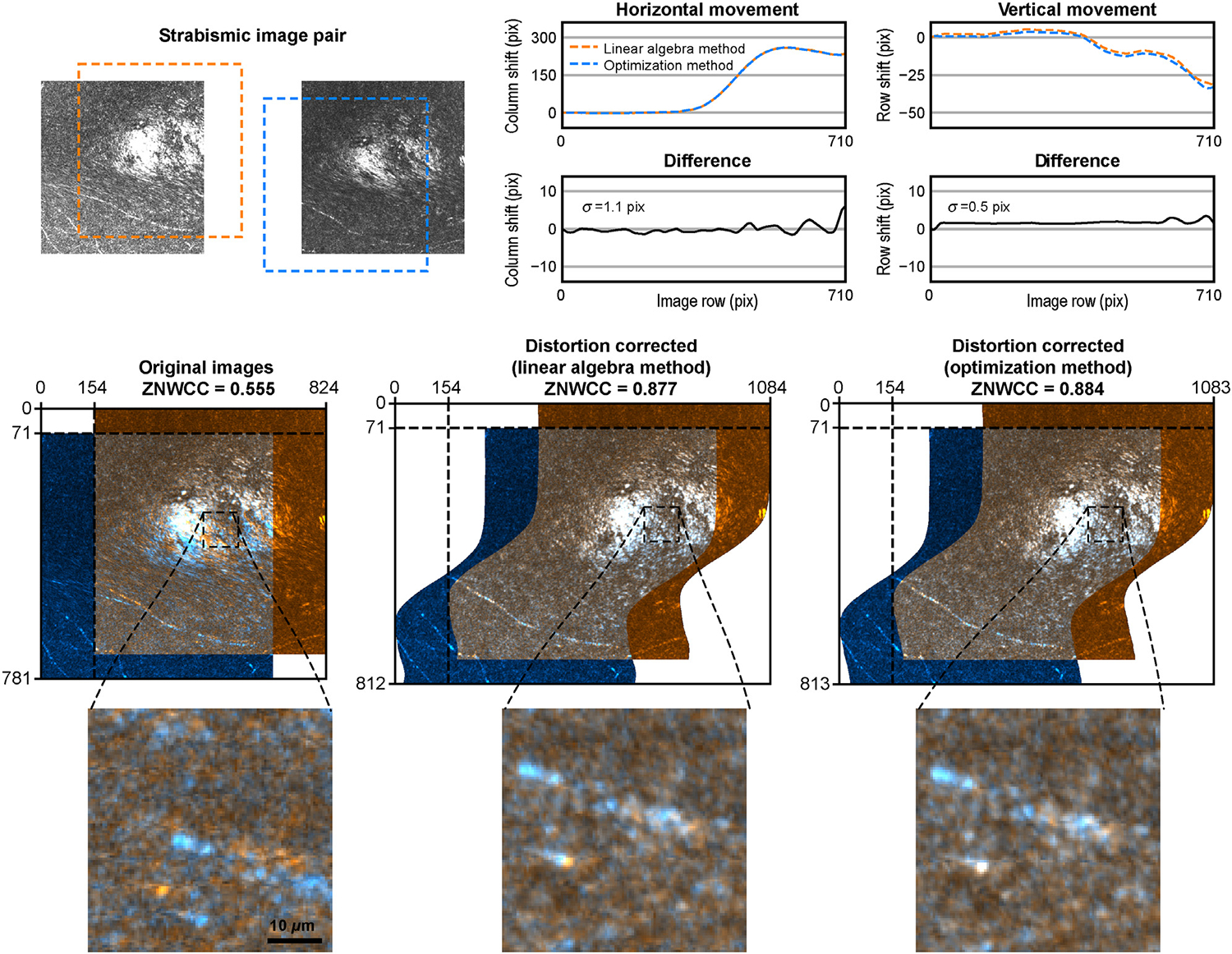
Strabismic reflectance image pair of a human foveal pit acquired with a point-scanning confocal ophthalmoscope (slow scanning from top to bottom), their respective outlines showing their relative positions (top left) and the estimated motion (top right). The images are shown before and after correction of the distortion caused by involuntary fixational eye movement, with the linear algebra approach first (one iteration with 5-pixel high strip, q=1), and then refined by three optimization iterations with 8.6-pixel sample spacing.

AOSLO retinal imaging provides an ideal test bed for evaluating strabismic image distortion correction because of the constant involuntary eye movements, including saccades, as illustrated in Figs. 8 and 9. The photoreceptor mosaic shown in Fig. 8 serves as a test object with no lines or vanishing directions such as those assumed in prior rolling shutter correction strategies.^23^ The mosaic of small bright image features over the dark background, the photoreceptor inner/outer segments, provides visual validation of the estimated motion over the overlapping region, despite substantial distortion at the top of the images. The difference plots show how the optimization approach improves the linear algebra solution by ~0.3 pixel overall (standard deviation across all image rows), increasing the ZNWCC by ~1.5%.

A particular AOSLO image pair of the bottom of the foveal pit in Fig. 9 exhibits a more severe distortion along both horizontal and vertical directions due to a strong saccade. In this case, the linear algebra estimation was performed in two iterations, due to the initial within-strip distortion degrading the motion estimation. The distortion correction in these images, despite having fewer and less well-defined structures, exemplifies that the proposed motion estimation is feature-agnostic and can cope with fairly severe motion distortion. Motion correction here also shows a substantial ZNWCC increase with both methods, with the optimization providing an improvement over the linear algebra solution of ~0.8 pixel standard deviation and 0.8% ZNWCC.

### 5.2 Dual-Rolling Shutter Camera Setup

**Fig. 10:**
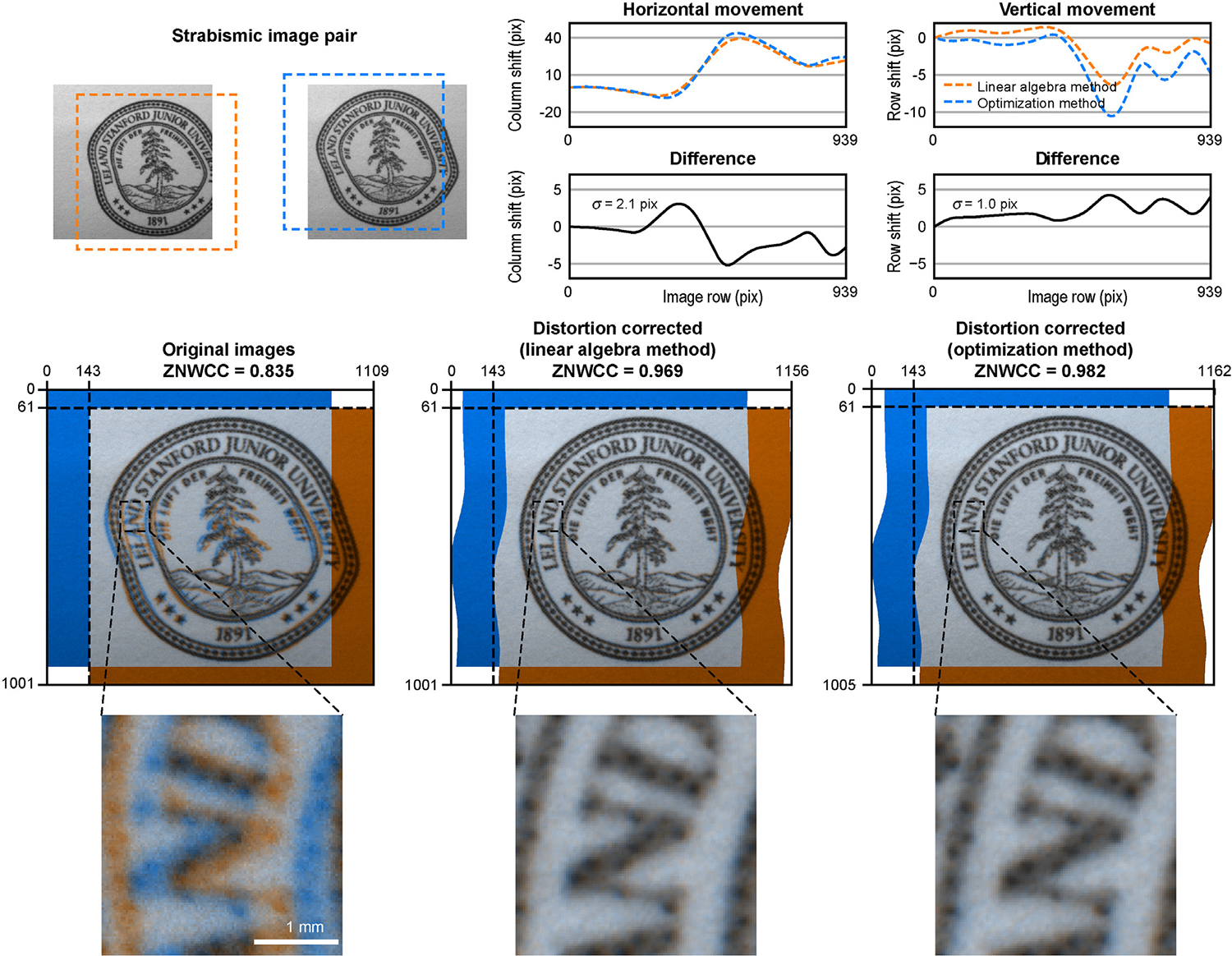
Strabismic image pairs of a Stanford logo acquired with two synchronous rolling shutter cameras (rolling from top to bottom) distorted by in-plane motion, with their respective outlines showing their relative positions (top left) and the estimated motion (top right). The images are shown before and after correction of the distortion caused by random movement of a mechanical oscillator, with the linear algebra approach first (one iteration with 21-pixel high strip, q=1), and then refined by three optimization iterations with 13.3-pixel sample spacing.

**Fig. 11:**
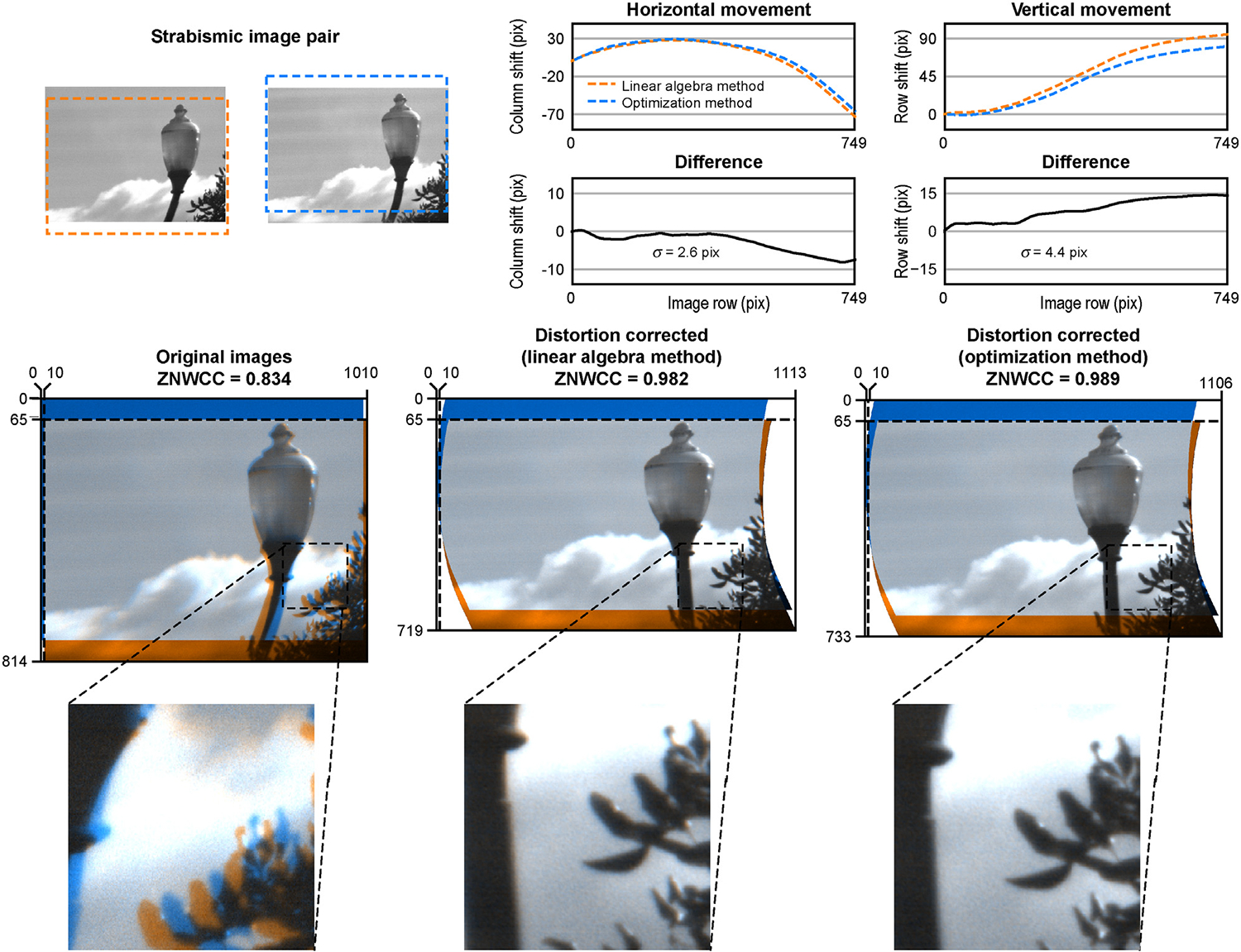
Strabismic image pairs acquired with two synchronous rolling shutter cameras (rolling from top to bottom) aiming through a window and distorted by in-plane motion, with their respective outlines showing their relative positions (top left) and the estimated motion (top right). The images are shown before and after correction of the distortion caused by random movement of a mechanical oscillator, with the linear algebra approach first (one iteration with 21-pixel high strip, q=1), and then refined by one optimization iteration with 93.75-pixel sample spacing.

The dual-camera setup was used to image a different university logo with random in-plane motion induced through a mechanical oscillator. The raw and processed images in Fig. 10 show correction of the motion distortion that is comparable to that in the AOSLO images, both with the linear algebra and optimization approaches. The same mechanical oscillator and optical setup with a different lens were used to image through a window to provide example images relevant to unmanned aerial vehicles and autonomous vehicles with within-frame velocity changes (see Fig. 11).

### 5.3 Timing

All strabismic image pairs were processed in a computer with an i9-14900K central processing unit (CPU) by Intel (Santa Clara, California, United States) running the operating system Windows 11 (Microsoft Corp., Redmond, Washington, United States), and a GeForce RTX 4070 graphics processing unit (GPU) by Nvidia (Santa Clara, California, United States). The motion estimation and correction software was written in the interpreted language Python, version 3.13, with the execution times shown in Table 1 below. The linear algebra approach was partially implemented for GPU but with no image strip pair processing parallelization or data transfer optimization. The optimization image processing approach was implemented to execute in CPU using a single thread.

## 6 Rotation and Scaling

**Fig. 12:**
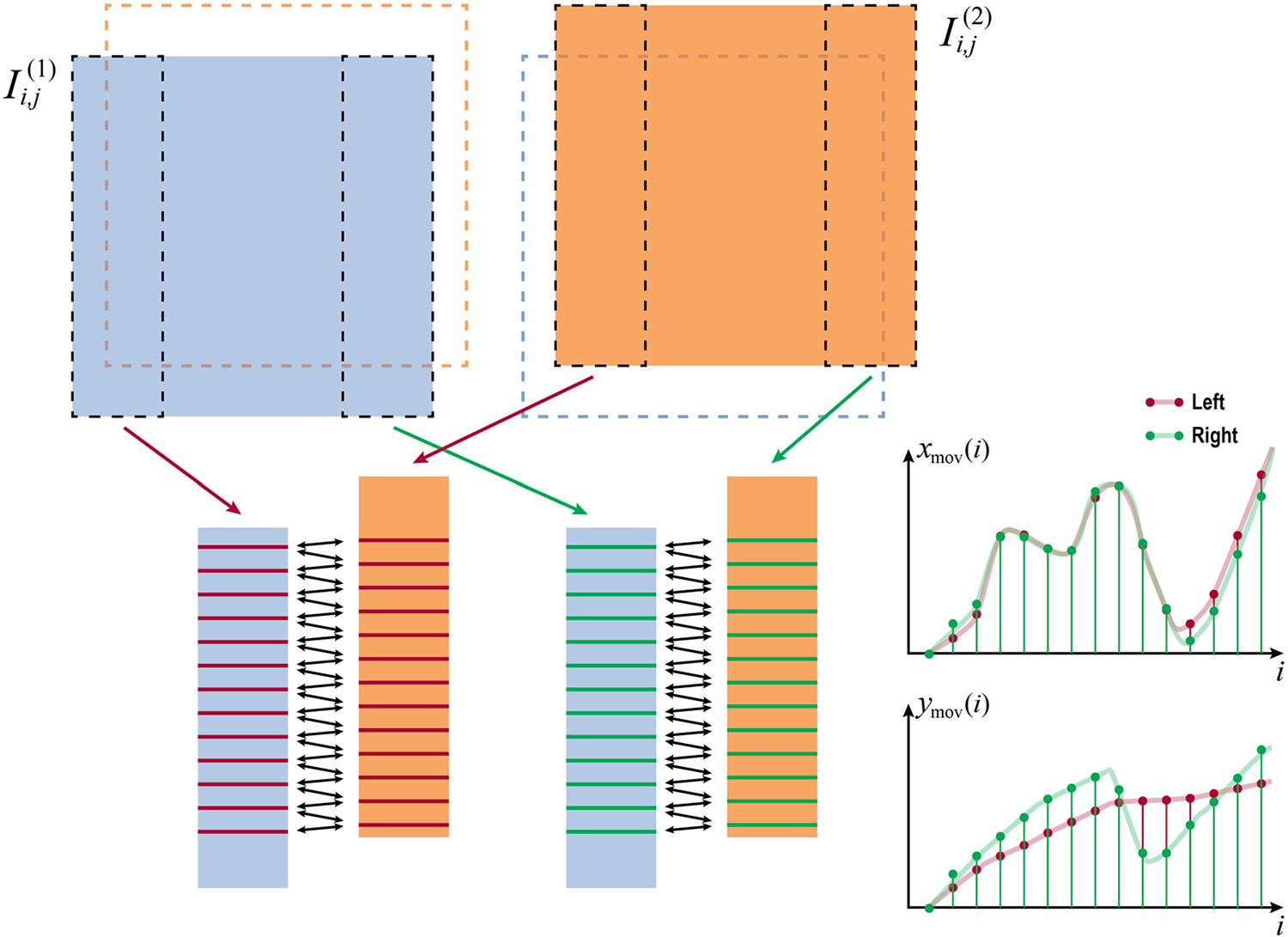
Modification of the method presented in Fig. 3 to estimate translations, rotations, and scaling changes during the simultaneous acquisition of strabismic image pairs (blue and orange rectangles) by co-registering image strip pairs for the left and right regions of interest with the plots depicting motion functions and their sampling. Note that all functions are set to start at zero.

Strabismic imaging can also be used to estimate and correct local (as opposed to global) rotation and changes in scaling. This can be achieved, for example, by cropping each image pair to create two pairs of narrow strabismic images, one capturing the left side of the field of view and the other capturing the right side, as depicted in Fig. 12. After using the algorithm described earlier to estimate the horizontal and vertical motion functions for the left and right sides of the images, local rotation and scaling changes can be estimated by comparing the rotation and changes in separation of corresponding left–right strip pair centers, respectively.^51^ The generalization of the optimization approach to estimate rotation and scaling changes requires allowing the $x_{\text{mov}}^{\prime }$ and $y_{\text{mov}}^{\prime }$ samples on the left and right sides of the image to vary independently, rather than just rigidly, with all the values in between those samples being linearly interpolated.

## 7 Summary

A dual-imaging method, termed strabismic imaging, was proposed to measure and correct distortion arising from in-plane object or scene motion in point-scanning instruments, line-scanning instruments, and rolling shutter cameras. The proposed motion estimation algorithms assume that image distortion is constant within each image row or column. This condition is satisfied by all line-scanning instruments and rolling shutter cameras and is a reasonable approximation for most point-scanning instruments. In addition to delivering the same frame rate as using a single channel, strabismic imaging allows motion correction in single image pairs and without additional information, and it is image feature-agnostic.

The estimation of the horizontal and vertical motion functions was formulated as a set of coupled linear equations corresponding to image strips along the (slow) scanning direction and as an optimization problem. The linear algebra approach is parallelizable and thus suitable for real-time implementation, whereas the optimization approach requires the use of sequential iterative algorithms that, although slower, can provide superior distortion correction. Both methods can be generalized to estimate and correct for object/scene rotation by comparing the motion functions of the left and right sides of the image. Also, the small temporal shift between the strabismic imaging of any given feature enables the measurement of fast intensity changes and velocity of moving objects within the field of view (e.g., blood cells^52^), also providing the ability to measure functional changes in live cells and tissues.

The ability to correctly estimate motion in the linear algebra approach depends on the spacing between the strips, which corresponds to the motion sampling, and the strip height, which can be thought of as a low-pass averaging filter. Also, the strip registration will fail (irrespective of the registration method) whenever the motion along the (slow) scanning axis is so severe that the features in one strip do not appear in the corresponding strip from the other strabismic image. The optimization approach can diverge or take long to converge whenever the motion is severe and, thus, could benefit from using the linear algebra approach to obtain an initial motion estimation, as demonstrated here. The accuracy of the motion estimation using both methods can be improved by upsampling the strabismic images, upsampling the strip ZNWCC function estimates, or increasing the number of optimization samples.

In scanning instruments, strabismic imaging can be implemented with an additional light detector and an adequate beam splitting element, without modifying the scanning optics or requiring an additional light source. Strabismic image pairs captured with point-scanning and dual-rolling shutter camera instruments were used to illustrate the motion estimation methods, which can also be applied to moving object(s) on a stationary background, via a pre-segmentation step and using the ZNWCC for registration.

## Figures and Tables

**Table 1:** Strabismic imaging motion distortion estimation timing.

Figure No.	Image size (pix)	Linear algebra	Optimization
6	710 × 671	10 s (one iteration, 9-pix strip height)	242 s (three iterations, 8.6-pix sample spacing)
8	710 × 671	5 s (one iteration, 5-pix strip height)	258 s (three iterations, 8.6-pix sample spacing)
9	710 × 671	24 s (two iterations, 21-pix strip height)	826 s (five iterations, 8.6-pix sample spacing)
10	940 × 966	67 s (one iteration, 21-pix strip height)	439 s (three iterations, 13.3-pix sample spacing)

## Data Availability

Software and data underlying the results presented in this paper are not publicly available at this time but may be obtained from the authors upon reasonable request.
